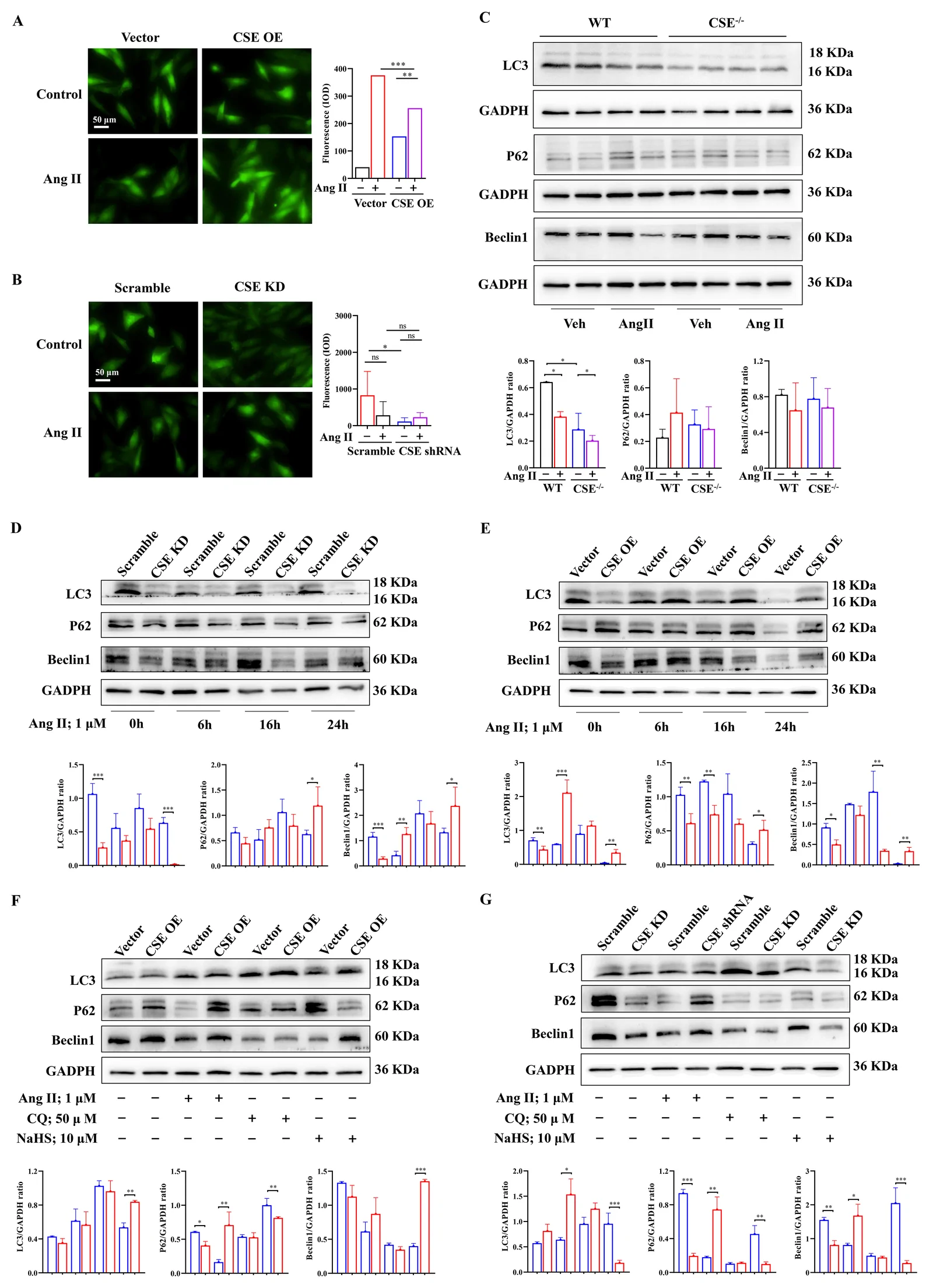# The ATG4 isotype is a key regulator in CSE/H_2_S-mediated mitophagy in cardiac hypertrophy

**DOI:** 10.1016/j.gendis.2026.102098

**Published:** 2026-02-16

**Authors:** Yijun Xin, Zhaoji Yuan, Yong Zhang, Xiaofei Qiao, Xiangdong Wang, Siying Li

**Affiliations:** a Metabolism and Disease Research Center, Central Hospital Affiliated to Shandong First Medical University, Jinan, Shandong 250013, China; b Shandong Institute of Endocrinology and Metabolic Diseases, Shandong First Medical University, Jinan, Shandong 250062, China; c Department of Cell Biology, School of Basic Medical Sciences, Shandong University, Jinan, Shandong 250012, China

Cardiac hypertrophy, which involves cellular oxidative stress, mitochondrial dysfunction, and even cell death, subsequently progresses to heart failure. The initiation of autophagy often accompanies this process to clear protein aggregates and damaged mitochondria. This study demonstrated that cystathionine γ-lyase (CSE), an enzyme responsible for the endogenous hydrogen sulfide (H_2_S) production, plays a regulatory role in cardiac hypertrophy primarily through the regulation of autophagy-related gene 4a (ATG4A), thereby influencing autophagosome formation and affecting the process of mitophagy. We demonstrate that CSE deficiency exacerbates angiotensin II (Ang II)-induced cardiac dysfunction and mitochondrial damage, while CSE overexpression (OE) is protective. Mechanistically, we show that the cardioprotective effects of CSE/H_2_S are specifically mediated through the regulation of the autophagy-related protein ATG4A, but not other ATG4 isoforms (B, C, D). ATG4A was essential for maintaining LC3 lipidation and PINK1/Parkin-mediated mitophagy.^1^ Our findings unveil a novel pathway where CSE/H_2_S signaling preserves mitochondrial quality control via ATG4A, highlighting a potential therapeutic target for mitigating cardiac hypertrophy.

Following four weeks of Ang II infusion, CSE knockout (CSE^−/−^) mice exhibited significantly exacerbated cardiac dysfunction compared with wild-type controls, as indicated by increased ventricular dimensions and impaired fractional shortening (Fig. S1A–S1C; Table S1). Histological analysis confirmed greater cardiac hypertrophy and fibrosis in CSE^−/−^ mice post-Ang II treatment (Fig. S1D–S1I). Expression of hypertrophy markers (ANP, BNP, α-MHC, β-MHC, and SERCA2) was markedly elevated in CSE^−/−^ hearts (Fig. S1J; Table S2). *In vitro*, Ang II treatment increased cardiomyocyte size in control cells (Vector, Scramble), an effect blunted by CSE OE and amplified by CSE knockdown (CSE KD) (Fig. S1K–S1M). Moreover, Ang II induced a significant increase in mitochondrial reactive oxygen species and loss of mitochondrial membrane potential in control cardiomyocytes. CSE OE mitigated this damage, while CSE KD exacerbated it (Fig. S1N, S1O).

CSE is a key enzyme involved in the endogenous production of H_2_S.^2^ CSE OE maintained H_2_S levels after Ang II challenge, while CSE KD further reduced them (Fig. S2A and S2B). In particular, H2S, as a crucial signaling molecule, has been shown to influence autophagy, a cellular process vital for maintaining cellular homeostasis and function.^3^ Western blotting analysis revealed that Ang II impaired autophagy, shown by reduced LC3-II and accumulated P62, with this impairment being more severe in CSE^−/−^ hearts (Fig. S2C) and in CSE KD cardiomyocytes (Fig. S2D). CSE OE rescued autophagic activity (Fig. S2E). Chloroquine chase assays confirmed that Ang II inhibited autophagic flux in control cells, which was restored by CSE OE or H_2_S donor (NaHS) treatment and worsened by CSE KD (Fig. S2F and S2G).

The PINK1–Parkin pathway, a key mediator of mitophagy, was down-regulated by Ang II in control cells. CSE OE maintained PINK1 and Parkin protein levels, whereas CSE KD caused a further significant reduction (Fig. 1A). Consistent with *in vitro* data, cardiac tissue from Ang II-treated CSE^−/−^ mice showed a lower PINK1/Parkin ratio than wild-type mice (Fig. 1B). The proteolysis of ATG4, a cysteine protease, leads to LC3 conjugation to phosphatidylethanolamine on the membrane, which is essential for autophagosome biogenesis. In mammalian cells, there are four homologous genes of ATG4. Among the four ATG4 isoforms, only ATG4A protein levels were significantly decreased by Ang II treatment in control cardiomyocytes. CSE OE prevented this decrease, and CSE KD resulted in even lower basal ATG4A levels (Fig. 1C). Levels of ATG4B, C, and D were unchanged. These results indicate that CSE is a potent regulator of ATG4A expression and thereby contributes to autophagosome formation. To establish causality, we modulated ATG4A directly. ATG4A OE enhanced LC3 lipidation and PINK1/Parkin expression, promoting mitophagy. Conversely, ATG4A KD reduced LC3-II and suppressed the PINK1/Parkin pathway, impairing mitophagy (Fig. 1D).

**Figure 1:**
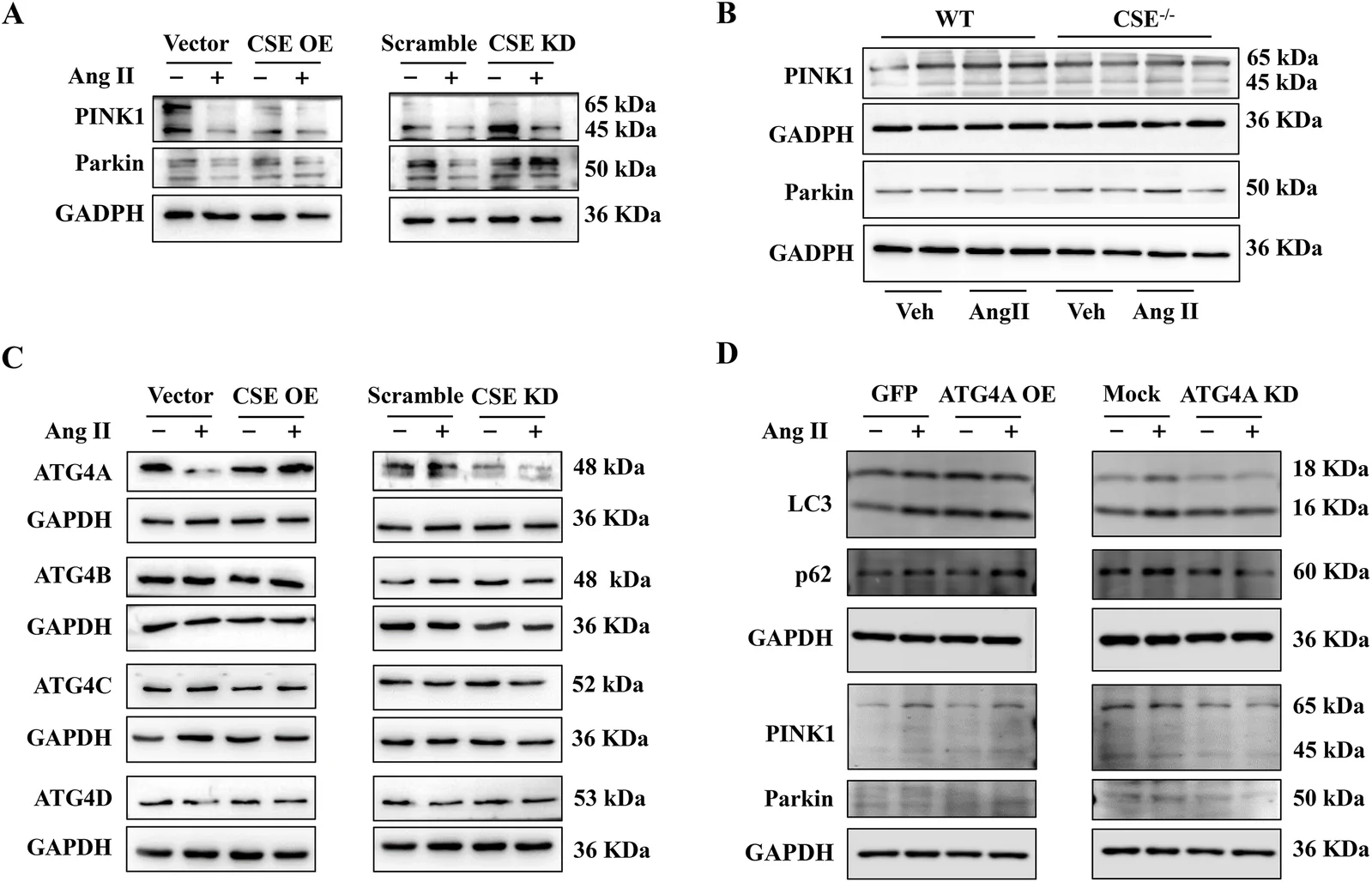
ATG4 regulates CSE/H_2_S-mediated mitophagy in angiotensin II (Ang II)-induced heart failure. **(A)** CSE overexpression (CSE OE) or CSE knockdown (CSE KD) in cardiomyocytes (H9C2) was constructed by lentivirus transfection. Cardiomyocytes were treated with Ang II (1 μM) for 24 h, and the mitophagy markers, PINK1 and Parkin, were examined by Western blotting in cardiomyocytes with CSE OE or KD. **(B)** The cardiac hypertrophy was induced by Ang II administration in wild-type mice (WT) and CSE knockout (CSE^−/−^) mice, and the normal saline group (Vehicle, Veh) was used as the control group. PINK1 and Parkin were tested in heart tissue after Ang II treatment in WT and CSE^−/−^ mice. **(C)** The four isotypes of ATG4 (ATG4A, ATG4B, ATG4C, and ATG4D) were examined by Western blotting in cardiomyocytes with CSE OE or KD. **(D)** LC3, P62, PINK1, and Parkin protein expression in ATG4A OE (ATG4 OE) or KD (ATG4 KD) under Ang II treatment in cardiomyocytes. All experiments were repeated in triplicate.

In summary, this study elucidated the impact and mechanism of CSE/H_2_S on cardiac hypertrophy by modulating mitophagy, with ATG4A identified as a pivotal regulator. We propose that H_2_S may regulate ATG4A via its well-established antioxidant properties, reducing oxidative inactivation of the protease, or through direct post-translational modification such as persulfidation, which can alter protein function. Critically, it has been reported that the cysteine protease ATG4 is a bona fide physiological target for H_2_S-mediated control of autophagy, which is regulated by persulfidation at its catalytic cysteine in *Arabidopsis*.^4^ This finding is further reinforced by the established role of ATG4 as a redox sensor within the autophagy machinery, as evidenced by its regulation via a reversible disulfide bond in yeast.^5^ Together, these studies demonstrate that the ATG4 protease family is inherently susceptible to functional modulation via cysteine-based redox modifications, thereby providing a robust mechanistic foundation for our proposed model. The therapeutic potential of H_2_S donors is limited by pharmacokinetic challenges, such as transient bioavailability. Our finding that ATG4A is a redox-sensitive component of the autophagy machinery reveals a more viable therapeutic target. Direct pharmacological targeting of ATG4A could thus offer a more precise and stable means to achieve the therapeutic benefits of H_2_S signaling in cardiovascular diseases, with improved pharmacokinetic properties and potentially fewer off-target effects.

This study has several limitations. First, we used a whole-body CSE^−/−^ model and a single hypertrophic stimulus (Ang II). Systemic effects, such as blood pressure changes, or contributions from other cell types (*e.g.*, fibroblasts) cannot be excluded. Future studies employing cardiomyocyte-specific knockout models and alternative pressure-overload models like transverse aortic constriction will confirm the cell-autonomous nature of this pathway. Second, while we implicate the PINK1–Parkin pathway, causal experiments using Parkin knockout models are needed to definitively establish its requirement. Finally, a more comprehensive analysis of mitochondrial function (respiration, ATP production) would deepen the insight into how H_2_S-ATG4A signaling preserves metabolic homeostasis.

## CRediT authorship contribution statement

**Yijun Xin:** Methodology. **Zhaoji Yuan:** Investigation, Formal analysis. **Yong Zhang:** Investigation, Formal analysis. **Xiaofei Qiao:** Methodology. **Xiangdong Wang:** Writing – review & editing. **Siying Li:** Writing – original draft, Supervision, Resources, Funding acquisition, Conceptualization.

## Ethic declaration

The animals were treated in accordance with the Guide for the Care and Use of Laboratory Animals, and all protocols were approved by the Ethics Committee of Shandong Institute of Endocrinology and Metabolic Diseases (20221101).

## Data availability

The data are available within the article or its Supplementary materials.

## Funding

This research was funded by the 10.13039/501100001809National Natural Science Foundation of China (No. 81800353).

## Conflict of interests

The authors declared no conflict of interests.

## Figures and Tables

**figs1:**
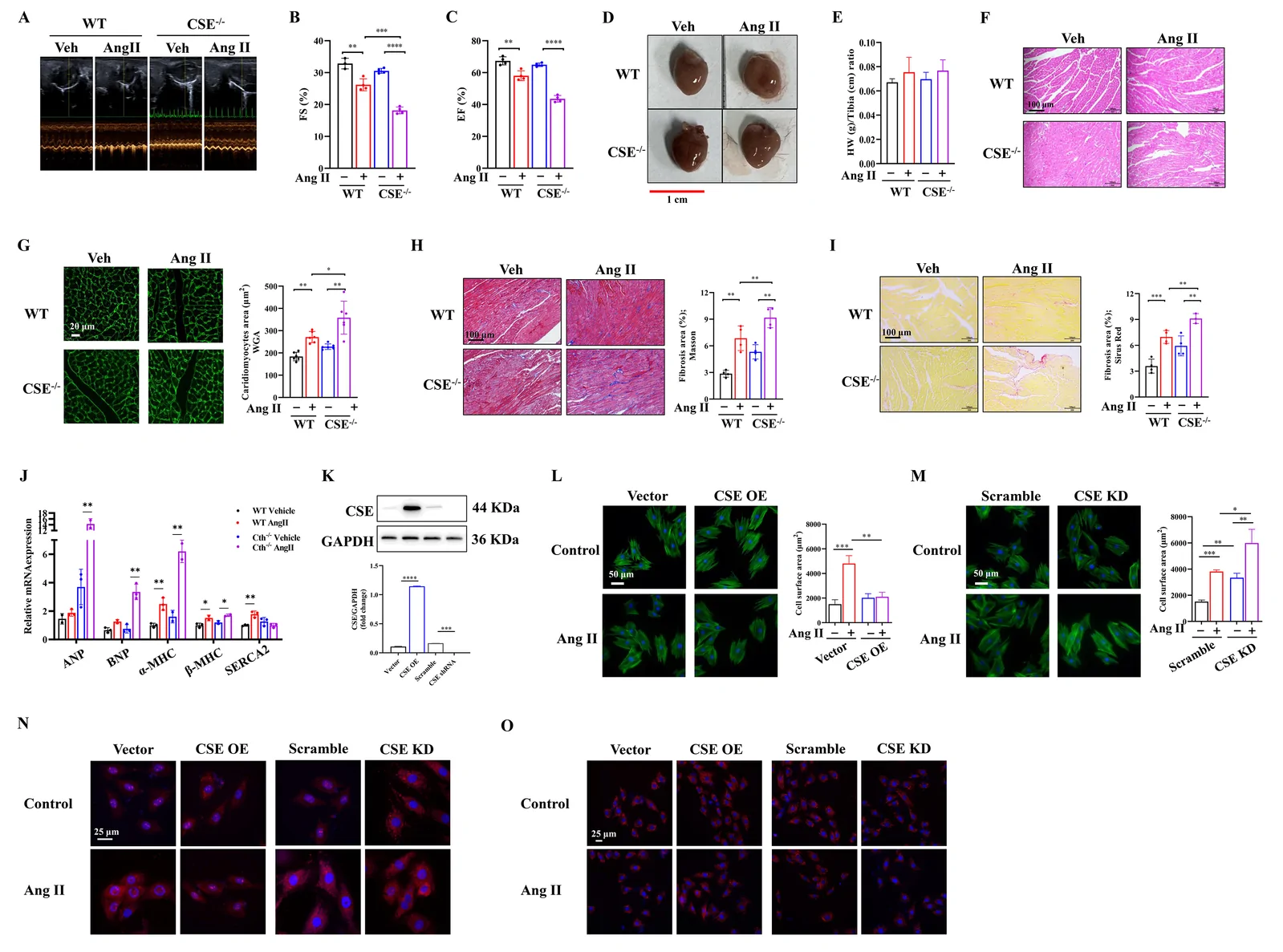


**figs2:**