# Supplementary material for: The ATG4 isotype is a key regulator in CSE/H2S-mediated mitophagy in cardiac hypertrophy
Source: Genes Dis. 2026 Feb 16;14(1):102098. doi: 10.1016/j.gendis.2026.102098 (PMC13545358; doi:10.1016/j.gendis.2026.102098)
Supplement: Supplementary file 1 [file mmc1.docx]

**Materials and Methods**

**Reagents**

Angiotensin II (A9525, Sigma-Aldrich), Sodium hydrosulfide hydrate (161527, Sigma-Aldrich), chloroquine phosphate (HY-17589, Med Chem Express) were used in this study.

**Cell Culture**

H9C2 rat cardiac fibroblasts (Cat. No. GNR 5) were purchased from the National Collection of Authenticated Cell Cultures, Chinese Academy of Sciences. Cells were cultured in Dulbecco's Modified Eagle's Medium (DMEM, Catalog No. 30-2002, Gibco) containing 10% fetal bovine serum (FBS). Cells were Incubated at 37°C in a suitable incubator with a 5% CO_2_ in air atmosphere.

**Lenti-virus for construction of stable cell lines, and adenovirus infection**

The overexpression and knockdown lentivirus of CSE was purchased from Cyagen Biosciences. H9C2 cells were inoculated into 96-well plates for 3,000-5,000 cells each well, and the cell density reached 70% another day. Lentivirus with appropriate MOI (50) was added and put back into the incubator for further culture for 48 h to observe the infection efficiency. Suitable concentration of puromycin was added for screening. After screening, the cells were transferred to 10 cm dishes, and the selected single cells were transferred to 24-well plates for culture. The efficiency of overexpression and knockdown was detected by western blot. Once the polyclonal populations were growing well, cell culture was expanded to prepare for the follow-up experiment.

Atg4A gain or loss-of-function approaches were achieved by infecting the cells with adenovirus of GFP, Atg4A, scramble shRNA, or rat Atg4A shRNA (WZ Bioscience Inc.) as described above.

**Cardiac hypertrophy animal model (Angiotensin II-induced)**

CSE knockout (CSE^-/-^) mice was generated using CRISPR/Cas9 gene editing technology by Cyagen Biosciences. C57BL/6N wild-type (WT) littermates and CSE^-/-^ mice at 8 weeks of age were selected as experimental mice. Animals were treated in accordance with the Guide for the Care and Use of Laboratory Animals, and all protocols were approved by the Ethics Committee of Shandong Institute of Endocrinology and Metabolic Diseases (20221101).

The mouse cardiac hypertrophy model was established as previously described, briefly, AngII infusion at a rate of 1,000 ng/kg/min by using mini-osmotic pumps (Alzet 2004), which increases systolic blood pressure^30^. Normal saline (vehicle) was used as control group. Four weeks after AngII treatment, WT and CSE^-/-^ mice were assessed for cardiac function by echocardiography using VINNO 6 LAB (VINNO Corporation) with a 23 MHz high-frequency transducer.

**Pathology and immunohistochemistry**

Four weeks after AngII treatment, the mice hearts were harvested, weighed, and frozen in liquid nitrogen. The length of the tibias from the condyles to the tip of the medial malleolus was measured by micrometer calipers.

The morphology, hypertrophy and fibrosis of cardiac myocytes were observed in heart slices which embedded in paraffin. In paraffin sections (5 µm), the wax was dissolved with an organic solvent and the tissue slices were rehydrated before staining. Hematoxylin-eosin (H&E) staining was used to observe the morphology of the heart tissue. Masson’s trichrome staining was used to detect the fibrotic part of the heart tissue, and the blue area indicated the fibrotic part. Fluorescent wheat germ agglutinin (WGA, Alexa Fluor 488 conjugate, W11261, Thermo Fisher Scientific Inc.) was used to label cell membranes.

**RNA extraction and real-time polymerase chain reaction (RT-PCR)**

After 12 hours of starvation, cardiomyocytes were treated with 1 µM AngII for 24 or 48 hours. RNA was extracted and reverse transcribed to cDNA. Cardiomyocyte hypertrophy-related genes, atrial natriuretic peptide (ANP), B-type natriuretic peptide (BNP), alpha-myosin heavy chain (α-MHC), beta-myosin heavy chain (β-MHC), and sarcoplasmic/endoplasmic reticulum calcium ATPase 2 (SERCA2) were detected by RT-PCR (Table 1).

**Cytoskeletal protein staining of cardiac cell**

Cytoskeletal protein staining was performed using Alexa Fluor™ 488 phalloidin (A12379, invitrogen™). Phalloidin storage solution (100×) was prepared by adding 500 μL DMSO to the powder and stored at -20℃. Before use, 100 μL PBS was added to 1 μL storage solution to make a working solution. The CSE overexpression (CSE OE) and knockdown (CSE KD) cardiomyocytes and the control cardiomyocytes were inoculated into the culture plate, and the cardiomyocytes were starved for 12 h. After 24 h of treatment with AngII (1 μM), the hypertrophy of cardiomyocytes was detected by phallodin fluorescence staining. The phallodin working solution was added to stain cells at room temperature for 30 minutes, and an anti-fluorescence quencher containing DAPI (P0131, Beyotime Biotechnology) was added. Fluorescence images were captured using a DeltaVision ultrahigh-resolution microscope (GE Healthcare, Issaquah, WA, USA). Images were analysed using ImageJ software (version 1.41, National Institutes of Health).

**H_2_S staining**

After the treatment of the cells in the 24-well plate, the cells were cleaned with preheated PBS for 3 times. Subsequently, a working solution of WSP-1 (50 mM, H_2_S fluorescence probe) (Shanghai Maokang Biotechnology Co., Ltd.) was added to the cells, and incubate at 37℃ for 30 min in the dark. Finally, the preheated medium was added to maintain the cell state and photographed under a fluorescence microscope (IX73, Olympus). The green fluorescence intensity represents the level of H_2_S.

**MitoSox Staining**

The CSE OE and CSE KD cardiomyocytes and the control cardiomyocytes were inoculated into the culture plate, and the cardiomyocytes were starved for 12 h. After 24 hours of treatment with AngII (1 μM), the myocardial cells were stained with CellROX Green dye to observe the Mitochondrial Superoxide level in the myocardial cells. Briefly, 5 mM MitoSox Red reagent (M36008, Thermo Fisher Scientific Inc.) were added into HBSS buffer solution and dilute it to 5 μM working solution, and preheat it away from light. Preheat PBS to clean the treated cells in the 24-well plate, add MitoSox working solution, and incubate in an incubator for 10 min at 37℃ away from light. Preheat PBS to clean the cells for 3 times, and add Hoechst 33342 (62249, Thermo Scientific™) to incubate and stain the cells for 5 min in the dark. Preheated PBS was used to clean the cells for 3 times, and the preheated medium was added to maintain the state of the cells. Photos were taken under DeltaVision ultrahigh-resolution microscope. The red fluorescence intensity represents the mtiochondria ROS (mtROS) level.

**Western Blot**

In CSE overexpression (CSE OE) and knockdown (CSE KD) cardiomyocytes and control cardiomyocytes After 24 hours of treatment with 1 μM AngII, the protein was extracted, and SDS-PAGE was used to detected protein expression by Western Blot. The antibodies were purchased as follows: Gamma Cystathionase antibody (Proteintech Cat# 12217-1-AP), Anti-LC3 antibody ((Sigma-Aldrich Cat# L8918), P62/SQSTM1 antibody (Proteintech Cat# 55274-1-AP), Beclin 1 antibody (Proteintech Cat# 66665-1-Ig, RRID:AB_2882020), PINK1 antibody (Proteintech Cat# 23274-1-AP), PARK2/Parkin antibody (Proteintech Cat# 14060-1-AP), Atg4A (D62C10) Rabbit mAb (Cell Signaling Technology Cat# 7613), ATG4B antibody (Cell Signaling Technology Cat# 5299), Atg4C antibody (Affinity Biosciences Cat# DF6259), ATG4D antibody (Proteintech Cat# 16924-1-AP).

**Statistical analysis**

All the data in this experiment are measurement data. If the data conform to normal distribution, the independent sample t test is used for analysis; if the data do not conform to normal distribution, the non-parametric rank sum test is used for analysis. And use GraphPad Prism 9.0 for image processing. P < 0.05 indicates statistically significant difference.

**Table S1. Echocardiography in WT and CSE^−/−^ mice after AngII treatment.**

|  |  | **WT** | |  | **CSE^-/-^** | |
| --- | --- | --- | --- | --- | --- | --- |
|  |  | **Vehicle** | **Ang II** |  | **Vehicle** | **Ang II** |
| **IVSd** | mm | 0.78±0.05 | 0.62±0.03 |  | 0.67±0.12 | 0.63±0.12 |
| **LVIDd** | mm | 3.93±0.04 | 4.41±0.24 |  | 4.14±0.16 | 4.31±0.25 |
| **LVPWd** | mm | 0.83±0.04 | 0.87±0.19 |  | 0.91±0.25 | 0.94±0.31 |
| **IVSs** | mm | 0.97±0.23 | 0.92±0.09 |  | 0.96±0.2 | 1.02±0.3 |
| **LVIDs** | mm | 2.91±0.09 | 3.26±0.25 |  | 2.88±0.11 | 3.52±0.18 |
| **LVPWs** | mm | 1.23±0.02 | 1.04±0.26 |  | 1.13±0.33 | 1.08±0.32 |
| **EF(M-Teich)** | % | 67.37±2.52 | 58.05±3.11 |  | 64.94±1.12 | 43.58±2.06 |
| **%FS(M)** | % | 41.15±16.65 | 26.2±1.89** |  | 30.56±0.77 | 18.15±1.07**** |
| **HR** | bpm | 475.88±44.6 | 503.25±47.03 |  | 472.25±42.92 | 438.25±89.71 |
| **BW** | g | 24.88±0.94 | 26.7±0.42 |  | 24.13±2.09 | 25.85±0.83 |

LVPW; d: left ventricular posterior wall thickness diastolic; LVPW; s: lef tventricular posterior wall thickness systolic; FS: fractional shortening; LV Vol; d: left ventricular volume; diastolic; LV Vol; s: left ventricular volume; systolic; HR: heart rate. * P<0.05; ** P<0.01; ***P<0.001; ****P<0.0001.

**Table S2. Murine Primers for qPCR**

| **Primers** | **Gene access#** | **Forward (5’-3’)** | **Reverse (3’-5’)** |
| --- | --- | --- | --- |
| **ANF** | NM_008725.2 | CATCACCCTGGGCTTCTTCCT | TGGGCTCCAATCCTGTCAATC |
| **BNP** | NM_008726.4 | GCGGCATGGATCTCCTGAAGG | CCCAGGCAGAGTCAGAAACTG |
| **α-MHC** | NM_010856.4 | CCAATGAGTACCGCGTGAA | ACAGTCATGCCGGGATGAT |
| **β-MHC** | NM_080728.2 | ATGTGCCGGACCTTGGAA | CCTCGGGTTAGCTGAGAGATCA |
| **SERCA2a** | NM_009722.3 | CCATCTGCTTGTCCATGTCACT | CAAATGGTTTAGGAAGCGGTTACT |
| **GAPDH** | XM_001479371.4 | ATGTTCCAGTATGACTCCACTCACG | GAAGACACCAGTAGACTCCACGACA |

**Figure Legend**

**Fig. S1 The effect of CSE on cardiomyocyte hypertrophy and heart failure**

The cardiac hypertrophy was induced by Angiotensin II (Ang II) administration in wild-type mice (WT) and CSE knockout (CSE^-/-^) mice, and normal saline group (Vehicle) was used as control group. **(A)** Representative images of the echocardiograph at 4 weeks after AngII administration in WT and CSE^-/-^ mice. The cardiac functional indicators: left ventricular brachyaxis **(B)** FS (%): shortening rate, and **(C)** EF (%): ejection fraction, were measured. **(D)** Cardiac morphology and size **(E)** and heart weight /Tibia ratio (HW/Tibia) of mice were recorded 4 weeks after AngII administration. The mouse heart was sliced and **(F)** H&E, **(G)** WGA staining, **(H)** Masson staining, **(I)** Sirius Red Staining were stained to observe the pathological changes. **(J)** Biomarkers indicative of heart failure, such as ANP, BNP, α-MHC, β-MHC, SERCA2, were measured by RT-PCR. **(K)** CSE overexpression (CSE OE) or knockdown (CSE KD) in cardiomyocytes (H9C2) was constructed by lenti-virus transfection, and CSE protein expression was examined by western blot. Cardiomyocytes were treated with Ang II (1 μM) for 24h, Fluor 488 phalloidin was stained to examine the hypertrophy of cardiomyocytes with **(L)** CSE OEor **(M)** CSE KD. *P<0.05; ** P<0.01; ***P<0.001.

**Fig. S2** The effect of CSE/H_2_S on autophagy and autophagy flux

The H_2_S expression was stained by WSP-1 probe in cardiomyocytes with **(A)** CSE OE or **(B)** CSE KD with shRNA. Scale bar: 50 μm. **(C)** The biomarkers of autophagy, LC3, P62 and Beclin1, were tested by Western blot in AngII-treated WT and CSE^-/-^ mice. The autophagy level was examined in different time points (0, 6, 16, 24 h) after AngII treatment in cardiomyocytes with **(D)** CSE OE or **(E)** CSE CSE KD. Cardiomyocytes were treated with CQ or NaSH before harvest, in order to determine the autophagic flux both in cardiomyocytes with **(F)** CSE OE or **(G)** CSE KD. All experiments were repeated triplicate. *P<0.05; ** P<0.01; ***P<0.001.
